# Trends in the Epidemiology of Leishmaniasis in the City of Barcelona (1996–2019)

**DOI:** 10.3389/fvets.2021.653999

**Published:** 2021-04-26

**Authors:** David Palma, Lilas Mercuriali, Jordi Figuerola, Tomás Montalvo, Rubén Bueno-Marí, Joan-Pau Millet, Pere Simón, Eva Masdeu, Cristina Rius

**Affiliations:** ^1^Agència de Salut Pública de Barcelona (ASPB), Barcelona, Spain; ^2^Centro de Investigación Biomédica en Red en Epidemiología y Salud Pública (CIBER-ESP), Madrid, Spain; ^3^Estación Biológica de Doñana (EBD-CSIC), Sevilla, Spain; ^4^Department of Research and Development (R&D), Laboratorios Lokímica, Valencia, Spain; ^5^Parasitology Area, Department of Pharmacy and Pharmaceutical Technology and Parasitology, University of Valencia, Valencia, Spain

**Keywords:** parasitology, leishmania, zoonosis, *Phlebotomus*, surveillance, One Health, public health surveillance, infectious disease

## Abstract

**Background:** Leishmaniasis is a neglected zoonosis produced by 20 different flagellated parasites of the *Leishmania* genus, a protozoan transmitted to humans and other vertebrates by the bite of dipteran insects of the *Phlebotominae* subfamily. It is endemic in Mediterranean countries and the number of cases is expected to increase due to climate change and migration. Prioritizing public health interventions for prevention and control is essential. The objective was to characterize the epidemiology and temporal trends in the incidence of human leishmaniasis in the city of Barcelona, between the years 1996 and 2019.

**Methods:** A population-based, analytical observational study among residents in the city of Barcelona was conducted of all the cases of leishmaniasis reported between 1996 and 2019 to the Public Health Agency. The epidemiological survey contains clinical, diagnostic, and epidemiological data, including contact with suspicious mammals or insects. Annual incidence-rates were calculated by sex, age, and country of origin. Chi-square tests were used to assess association between studied risk factors, periods of time and type of leishmaniasis.

**Results:** During the study period a total of 177 cases of leishmaniasis were reported in Barcelona, being 74.6% (*n* = 132) of the total cases in Spanish born, although within the foreign-born population the incidence was higher. Median age was 34 years (IQR = 10–48) and 121 (66.8%) were male. The main type was cutaneous (46%) followed by visceral (35.1%). The cumulative incidence was 0.47 per 100,000 inhabitants, with the highest incidence found in 2017 (1.60 per 100,000 inhabitants). A higher incidence was observed in the 0–4-year-old group (1.73 per 100,000 inhabitants), but increased during the study period for all age groups. There was an increase of foreign origin cases, and a decrease in the number of cases associated to any immunosuppression.

**Conclusion:** In Barcelona, leishmaniasis incidence continues to be higher in people under 5 years of age, and 25–64 years old males, but it has also increased in population from foreign country of birth. There is an increase of the cases since 2016, probably due to the changes in the notification system, increasing the diagnosis of cutaneous leishmaniasis. Improvements in the current surveillance system are needed. Notification of the disease, vector, and reservoir control activities are also essential for the control of the disease.

## Introduction

Leishmaniasis is a zoonosis produced by 20 different flagellated parasites of the *Leishmania* genus, a protozoan transmitted to humans and other vertebrates by the bite of dipteran insects of *Phlebotominae* subfamily (1, 2). The parasites that cause leishmaniasis are present in 98 countries around the world, causing between 700,000 and 1 million new cases annually, with an estimated 350 million people at risk of infection worldwide (1–4). There are three main clinical forms visceral leishmaniasis (VL), cutaneous leishmaniasis (CL), and mucocutaneous leishmaniasis (ML) (1, 4). VL is the most serious form of leishmaniasis and causes a systemic disease characterized by irregular episodes of fever, spleen and liver enlargement, weight loss and anemia. CL is the most frequent presentation and causes skin lesions that evolve from nodules to painless ulcers, which can leave long lasting scars. ML is the most uncommon form of leishmaniasis, and leads to the destruction of nasooropharyngeal mucosa, usually after years of the initial skin lesions. Diagnosis is established through direct observation or molecular detection of *Leishmania* parasites in tissue specimens, mainly from skin lesions, for cutaneous leishmaniasis, or from bone marrow, for visceral leishmaniasis, although serological tests could also be used in different situations (2). The wide diversity of *Leishmania* species is associated with a difference in the clinical presentation, routes of transmission (zoonotic vs. anthroponotic) and treatment resistance (2, 5). In this sense, VL is usually caused by *L. donovani* or *L. infantum*, while CL can be caused by *L. infanfum, L braziliensis*, or *L. tropica*, between others (2).

In urban, periurban and domestic environments, dogs (*Canis lupus familiaris*) are usually considered main reservoirs of the parasite, although synanthropic rodents (e.g., *Rattus norvegicus*), wild rabbits (*Oryctolagus cuniculus*), hares (*Lepus granatensis*), and cats (*Felis catus domesticus*) can also play a relevant role as reservoir host (6–12).

In endemic developing countries, leishmaniasis mainly affects people of lower socioeconomic status, in rural areas with poor housing conditions, with little or no access to health services (1, 13). Within the risk groups, visceral leishmaniasis can present non-specific symptoms in children, and can be fatal without adequate treatment (14). Although coinfection with HIV is considered a public health problem in various parts of the world (15), the introduction of highly effective antiretroviral treatment in developed countries since 1997 has helped to decrease the HIV coinfection (16), so nowadays it is a pathology more common in immunosuppression non-HIV-related (17).

Incidence of Leishmaniasis in Mediterranean countries and across Europe has been described on the rise (1). Autochthonous cases have been recently described in non-endemic areas, like Southern Germany (18), and the number of imported cases has increased in areas such as Sweden or London (19, 20). In Sweden, an increase in diagnoses has been observed since 2013, especially in those under 18 years of age and migrants, mainly from Syria and Afghanistan (19). In London, most diagnoses occurred in tourists returning from the Mediterranean region and soldiers returning from other territories where the pathogen is endemic (20).

Climate change is expected to extend the geographical distribution and seasonality of phlebotomine vectors (7, 21). In Morocco, the increase in minimum temperatures has increased the survival of the sandfly larvae through the winters, extending their activity season. This combined with the increase in human circulation and tourism is thought to be one of the main causes for dissemination of cutaneous leishmaniasis (22). Spain has also experienced an increase in cases, though this phenomenon has been attributed to multiple factors, including improvements in notification, the seasonality of sandflies (16) and a growing urbanization that favors the contact between reservoirs and humans (23).

Studies in different areas of Spain have reported seroprevalence varying from 4 to 35% in dogs, with a high proportion of them not showing any clinical signs of leishmaniasis (24, 25). *Phlebotomus perniciosus* and *Phlebotomus ariasi* are the main vectors of *Leishmania infantum* in the Iberian Peninsula (23, 26). Moreover, in Spain, leishmaniasis is a hypoendemic, mandatory notifiable disease since 1995 (27), but reporting is compulsory for every Autonomous Region (CCAA) through the National Surveillance Network since 2015 (28). During the 2005–2017 period, Spain had an incidence rate of 0.62 cases per 100,000 inhabitants (16, 25), with a heterogeneous distribution, mainly affecting the Mediterranean region (the Valencian Community, Catalonia, the Balearic Islands and Andalusia), except for the Community of Madrid, where a large community outbreak was identified in 2009 and 2010 linked to a newly identified reservoir (26). The objective of this study is to characterize the epidemiology and temporal trends in the incidence of human leishmaniasis in the city of Barcelona, between the years 1996 and 2019.

## Methodology

### Study Design and Population

A population-based, analytical observational study was conducted with all reported leishmaniasis cases between 1996 and 2019. Data were drawn from the registry of notifiable diseases of the Epidemiology Service of the Public Health Agency of Barcelona (ASPB). The registry includes cases individually notified to the Epidemiology Service by healthcare professionals and, since 2016, it also includes laboratory notifications to the Catalan Microbiologic Notification System (SNMC) (29).

### Variables

The registry of notifiable diseases contains the information included in the epidemiological surveys of cases carried out by public health nurses from the Epidemiology Service.

The epidemiological survey contains clinical, diagnostic, and epidemiological data, among others (Annex 1). The variables of interest included: age, sex, country of birth, date of onset of symptoms (onset, diagnosis, and notification data), type of leishmaniasis, history of immunosuppressive disease or immunosuppressive treatment, as well as history of contact with an infected dog or another animal suspected of infection and travel to an endemic country according to WHO's current guidelines at the time of the survey (30).

### Inclusion and Exclusion Criteria

All cases of leishmaniasis from patients who resided in the city of Barcelona and who met the case definition according to local guidelines were included (Annex 2). Cases were excluded if the patients resided in another city than Barcelona.

### Analysis

Annual incidence-rates and its confidence interval (95%) were calculated by sex, age, and country of birth, in relation to the municipal registry of the city of Barcelona (31). The microbiological confirmation and national surveillance system were modified in 2016 (28). Consequently, to test for temporal changes in the influence of these variables on the incidence of leishmaniasis, the study period was divided into three periods: 1996–2005 (*n* = 55), 2006–2015 (*n* = 52), and 2016–2019 (*n* = 70).

The risk factors obtained from the survey were analyzed by the three proposed periods and the clinical presentation of the leishmaniasis. Cases were classified according to the Available Family Income Index of the municipal district of residence (IRFD for “Index renda familiar disponible”), into disadvantaged (IRFD < 1) or favored (IRFD > 1) neighborhood (32). In 2019, 62.2% of Barcelona's population lived in disadvantaged neighborhoods (31). A chi-squared or a Fisher's exact test were performed, as appropriate, and a *p* < 0.05 was considered significant. All the analyses were done using the program STATA version 15.

## Results

**Figure 1:**
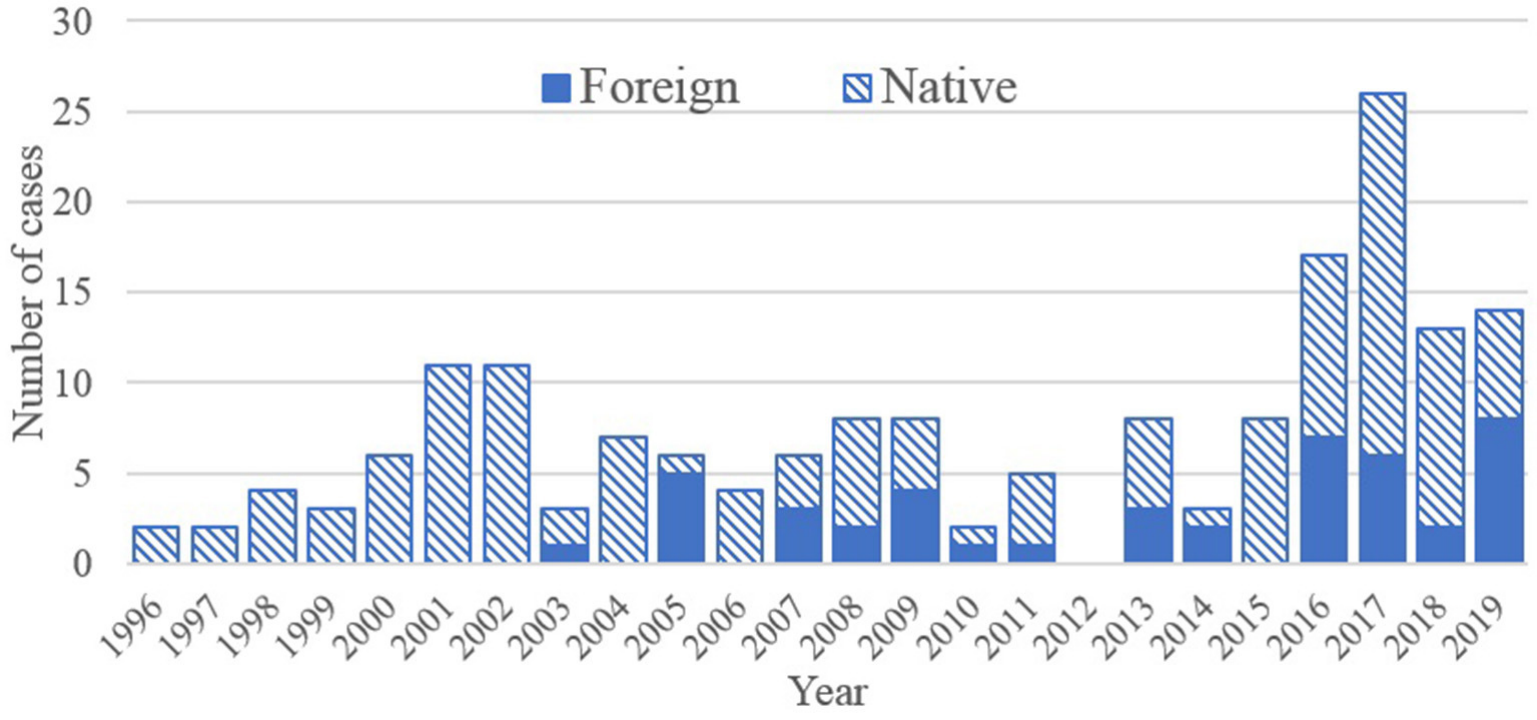
Total number of cases of leishmaniasis, in Spanish born population (native) and foreigner country of birth, in Barcelona between 1996 and 2019 (*n* = 177 cases).

Between 1996 and 2019, 177 cases of leishmaniasis were reported in Barcelona (Table 1). The median age was 34 years [interquartile range (IQR): 10–48]. 66.8% (*n* = 121) of the diagnoses occurred in men. According to origin, most cases occurred in native population (Table 1), however, a sustained increase in cases from foreign-born population were observed since 2003 (Figure 1). The main type of leishmaniasis was cutaneous (45.2%, *n* = 80), followed by visceral (34.5%, *n* = 61), and only 2.3% (*n* = 4) was mucocutaneous. 61.6% (*n* = 109) of the cases occurred in people who lived in disadvantaged municipal districts. 68.4% (*n* = 121) of the cases progressed to healing without sequel, 6.21% (*n* = 11) progressed with sequel, and 1.63% (*n* = 3) died (Table 1). The diagnosis were made by biopsy in 61% of the cases (*n* = 108), by a serological test in 21.5% (*n* = 38) and by culture in 19.4% (*n* = 21).

**Table 1:** Descriptive statistics of the leishmaniasis cases reported in Barcelona between 1996 and 2019 (*n* = 177).

	***n***	**%**
**Sex**		
Men	121	68.4%
Women	55	31.1%
**Country of origin**		
Native	132	74.6%
Foreign	45	25.4%
**Type of leishmaniasis**		
Visceral	61	34.5%
Cutanous	80	45.2%
Mucocutaneous	4	2.3%
Missing	32	18.1%
**Neighborhood's available family income index (AFII)**		
Disadvantaged (AFII < 1)	109	61.6%
Favored (AFII > 1)	57	32.2%
Missing	11	6.2%
**Evolution**		
Cured without sequelae (a)	121	68.4%
Cured with sequelae (a)	11	6.2%
Dead	3	1.7%
Lost to follow up (b)	42	23.7%

*(a) Sequelae defined by the surveyor's criteria*.

*(b) Of the 42 lost cases of “Evolution,” 9 cases were identified as “lost” by the person who carried out the survey, while the rest of the cases had no information*.

*In all the missing cases of the Type of leishmaniasis and Neighborhood, the value was not recorded in the epidemiological survey*.

**Figure 2:**
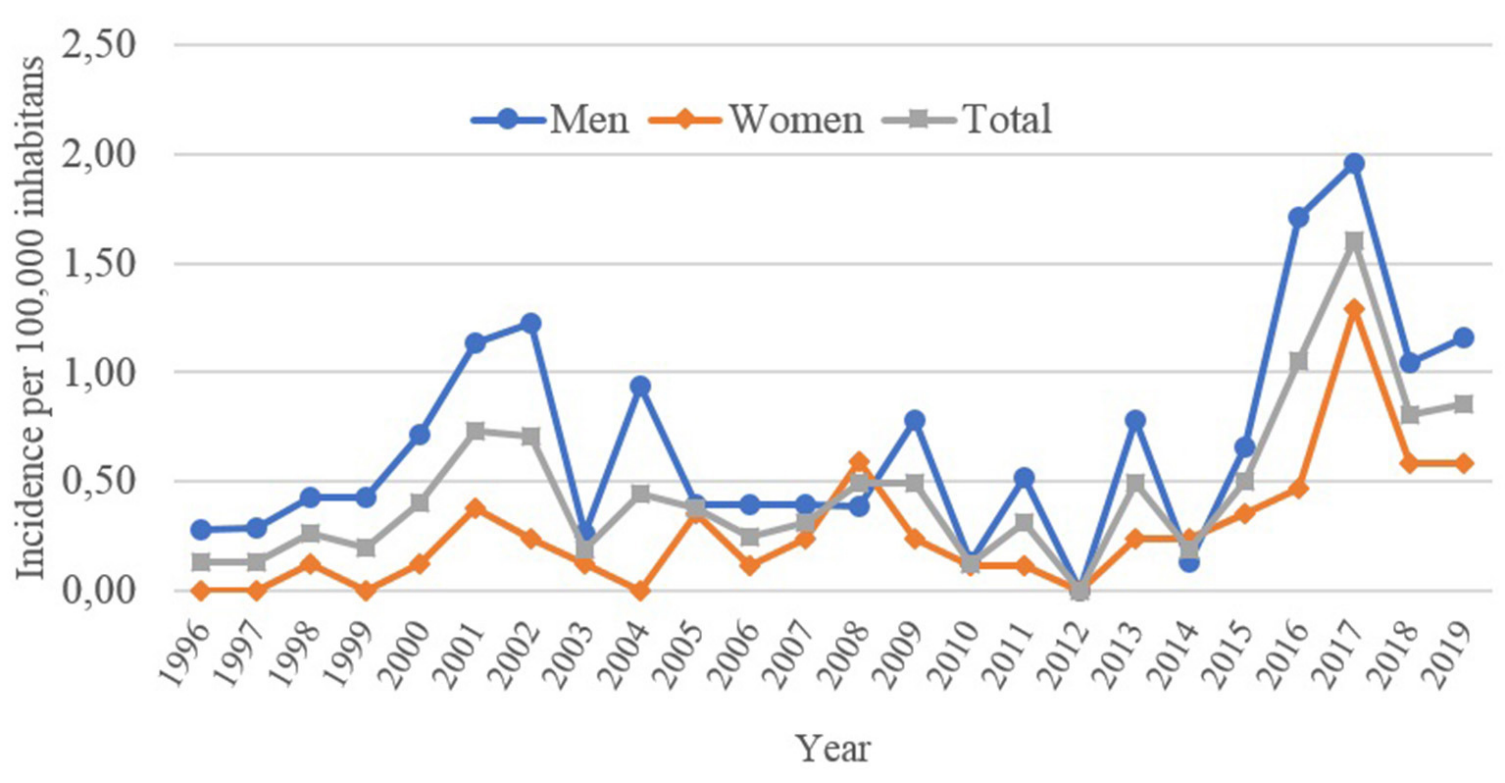
Annual incidence of leishmaniasis per 100,000 inhabitants, by complete sample and sexes. Barcelona, 1996–2019. (*n* = 177 cases).

The cumulative incidence during the study period was 0.47 per 100,000 inhabitants (Table 2), with the highest incidence found in 2017 (1.6 per 100,000 inhabitants) and the lowest in 2012 (Figure 2). Incidence was generally higher in men than women (0.68 vs. 0.27 per 100,000 inhabitants) and in foreign-born over Spanish-born population (0.74 vs. 0.41 per 100,000 inhabitants). Slight changes in incidence were observed between the first and second period (0.36–0.32 per 100,000), increasing significantly to 1.08 per 100,000 after 2016. An increase of the incidence was observed in all age groups, especially in the third period, with the higher incidence found in the 0–4-year-old group (1.73 per 100,000 inhabitants) (Table 2).

**Table 2:** Incidence of leishmaniasis per 100,000 inhabitants and confidence interval (95%), by complete study time and periods 1996–2005 (*n* = 55), 2006–2015 (*n* = 52), and 2016–2019 (*n* = 70), Barcelona.

		**1996–2005**	**2006–2015**	**2016–2019**	**Total**
**Sex**	Female	0.14 [0.06–0.22]	0.22 [0.12–0.32]	0.73 [0.44–1.02]	0.27 [0.19–0.34]
	Male	0.61 [0.43–0.79]	0.41 [0.27–0.55]	1.47 [1.04–1.89]	0.68 [0.56–0.80]
**Age**	0–4	2.00 [0.87–3.13]	1.15 [0.35–1.94]	2.59 [0.67–4.51]	1.73 [1.52–1.93]
	5–14	0.17 [0.09–0.24]	0.00	1.64 [0.56–2.71]	0.37 [0.15–0.59]
	15–24	0.16 [0.10–0.22]	0.14 [0.08–0.20]	1.03 [0.21–1.85]	0.28 [0.11–0.45]
	25–44	0.71 [0.47–0.95]	0.44 [0.26–0.62]	0.91 [0.49–1.33]	0.62 [0.48–0.76]
	45–64	0.11 [0.00–0.22]	0.35 [0.17–0.53]	0.70 [0.30–1.09]	0.31 [0.19–0.42]
	65 or more	0.03 [0.01–0.05]	0.15 [0.02–0.28]	1.23 [0.65–1.81]	0.30 [0.18–0.42]
**Country of origin**	Native	0.34 [0.24–0.44]	0.28 [0.19–0.37]	0.95 [0.68–1.22]	0.41 [0.34–0.48]
	Foreign	0.50 [0.09–0.90]	0.48 [0.24–0.72]	1.48 [0.88–2.08]	0.74 [0.52–0.96]
**Total**		0.36 [0.27–0.45]	0.32 [0.23–0.41]	1.08 [0.83–1.33]	0.47 [0.40–0.54]

During the three studied periods there was an increase of the cutaneous leishmaniasis in the second and third period (0–51.9 to 75.7%, *p* < 0.001), and a decrease of the visceral leishmaniasis in the final period (54.6 to 46.2% to a 10%, *p* < 0.001) (Table 3). Male sex accounted for over 80% of the cases in ages 25–64, without greater differences during the studied periods. Over 60% of cases occurred in disadvantaged neighborhoods during the three periods (*p* = 0.261). Leishmaniasis in foreign subjects increased over time, but only accounted for a third of the total cases in the last period. A significant decrease in cases related to any immunosuppression (due to disease or treatment) was observed (*p* < 0.001).

**Table 3:** Cases of leishmaniasis according to type of disease and risk factors, during the three studied periods.

	**1996–2005**	**2006–2015**	**2016–2019**	**Total**	***p-*value**
	***N* (%)**	***N* (%)**	***N* (%)**	***N* (%)**	
**Type of leishmania**					<0.001
Visceral	30 (54.6%)	24 (46.2%)	7 (10%)	61 (34.5%)	
Cutaneous	0	27 (51.9%)	53 (75.7%)	80 (45.2%)	
Mucocutaneus	1 (1.82)	0	3 (4.3%)	4 (2.3%)	
**Risk factors**					
Male sex by ages					
Male (0–4)	7 (58.3%)	4 (50%)	2 (28.6%)	13 (48.3%)	0.545 *f*
Male (5–14)	2 (100%)	0	5 (55.6%)	7 (63.6%)	0.491 *f*
Male (15–24)	1 (33.3%)	1 (50%)	3 (50%)	5 (45.5%)	1 *f*
Male (25–44)	30 (90.9%)	16 (72.7%)	14 (77.8%)	60 (82.2%)	0.202 *f*
Male (45–64)	4 (100%)	9 (64.3%)	11 (91.7%)	24 (80%)	0.149 *f*
Male (65 or more)	0	2 (40%)	10 (56.6%)	12 (50%)	0.640 *f*
Disadvantaged	31 (63.3%)	36 (75%)	42 (60.9%)	109 (65.7%)	0.261
neighborhood					
Foreign origin	6 (10.9%)	16 (30.8%)	23 (32.9%)	45 (25.4%)	<0.001
Immunosuppression	25 (45.5%)	18 (34.6%)	7 (10%)	50 (28.5%)	<0.001
Due to disease	24 (61.5%)	17 (32.7%)	6 (8.6%)	47 (29.2%)	<0.001
Due to treatment	12 (30.8%)	6 (11.5%)	5 (7.1%)	23 (14.3%)	0.009
Total cases	55 (100%)	52 (100%)	70 (100%)	177 (100%)	

*P-values presented with an italic f (f) when Fisher's exact test is performed*.

All types of leishmaniasis occurred mainly in Spanish born, men, and people from disadvantaged neighborhoods (Table 4). Visceral leishmaniasis was present in 40.9% of the cases that referred contact with an animal and 26% of the cases that referred intravenous drug use. A 33.8% of the cases who referred travel to an endemic country presented a cutaneous type of leishmaniasis (endemic country according to WHO's current guidelines at the time of the survey). Most of the visceral leishmaniasis required a hospital admission and it was the only leishmaniasis type related to a fatal outcome.

**Table 4:** Type of Leishmaniasis according to risk factors and evolution.

	**Visceral**	**Cutaneous**	**Muco cutaneous**	**Total**	***P*-value**
	***N* (%)**	***N* (%)**		***N* (%)**	**(a)**
			***N* (%)**		
**Risk factors**					
Foreign origin	12 (19.7%)	26 (32.5%)	0	45 (27.9%)	0.092 *f*
Male sex	43 (71.7%)	52 (65%)	4 (100%)	109 (68.1%)	0.491
Disadvantaged	39 (69.6%)	51 (64.6%)	3 (75%)	102 (75%)	0.911
neighborhood					
Immunosuppression	31 (50.8%)	8 (10%)	1 (25%)	50 (31.1%)	<0.001 *f*
Contact with animal	25 (40.9%)	9 (11.3%)	1 (25%)	35 (21.8%)	<0.001 *f*
Travel to endemic	8 (13.1%)	27 (33.8%)	0	35 (20.1%)	<0.001 *f*
country					
History of transplant	1 (1.6%)	1 (1.3%)	0	2 (1.2%)	^*^
History of alcoholism	8 (13.1%)	3 (3.8%)	1 (25%)	12 (6.9%)	<0.001 *f*
History of transfusion	5 (8.2%)	0	0	5 (2.9%)	^*^
Intravenous drug use	16 (26.2%)	0	0	16 (9.2%)	^*^
**Evolution**					
Hospital admission	57 (93.4%)	1 (1.3%)	1 (25%)	62 (32.6%)	
Cured without	51 (83.6%)	67 (83.8%)	3 (75%)	121 (68.4%)	
sequels					
Cured with sequels	4 (6.6%)	6 (7.5%)	1 (25%)	11 (6.2%)	
Death	2 (3.3%)	0	0	3 (1.7%)	
**Total**	61 (34.5%)	80 (45.2%)	4 (2.3%)	177 (100%)	

Barcelona, 1996–2019 (missing values = 32; 18.08%). P-values presented with an italic f (f) when Fisher's exact test is performed, and with an asterisk (*) when not performed due to cases with very low frequency.

Even if the current epidemiological survey does not systematically collect information on type of comorbidities, homelessness status, species of *Leishmania* or the type of animals that could have acted as a reservoir, it was possible to extract some of this information from the comments included by interviewers. Regarding comorbidities, it was observed that an 18.4% (*n* = 35) of the studied population had a diagnosis of HIV and two cases were described as Hepatitis C positive. Four cases were informed to be homeless. Seven cases were characterized as *Leishmania donovani*, and one case was described as *Leishmania braziliensis*.

## Discussion

The results of this study have shown an increase in the incidence of leishmaniasis in the city of Barcelona from 1996 to 2019. This increase only appears as statistically significant for the period 2016-2019. The establishment of the *Catalan Microbiologic Notification System* as a consequence of the decree 203/2015 (29), which requires mandatory notification of all identified cases by laboratories, is likely to have contributed greatly to the observed trend, together with other factors such as the improvement in diagnostic tools and possibly a real change in disease burden. The increase is observed in all ages, being greater in the younger population and male adults. The number of foreign-born cases has increased since 2003 while the number of cases associated to immunosuppression has decreased. There is also a change in the type of leishmaniasis in the three studied periods, with an increase of cutaneous leishmaniasis.

The cumulative incidence during these 24 years of observation was higher than previously reported in analysis of the same database (0.33 per 100,000 inhabitants between 1997 and 2014) (33) but lower than the reported incidence of Spain (16, 25). After the modification of the confirmation and surveillance system (28, 29), there was a significant increase of the total incidence probably due to an improved reporting, and because a possible increase in the clinical suspicion at primary care level.

Our study has shown that men between 25 and 64 years old present a higher incidence of leishmaniasis in all the studied periods. Previous studies have associated this correlation with the incidence of HIV in this group (33). However, the reasons why men of working age have a higher incidence of leishmaniasis are diverse, and cannot be exclusively related to this comorbidity, even when the data obtained through the epidemiological survey does not allow us to test for further causality. In this population, men accounted for 91.9% of the cases related to alcoholism, 81.3% of cases with a history of intravenous drug use and 70% of traveling to endemic countries. Studies have described a male bias in certain infectious diseases (34), including leishmaniasis (35), proposing physiological and behavioral theories. However, the evidence is not conclusive, and we cannot exclude that man are more exposed to infected vectors because of their social or working activities.

By age groups, there was a consistent high incidence in cases under 5 years of age, but also a rise in the cases over 65 years since 2001. In the younger population, the main causes are associated to a lack of effective immune response (14, 33). In our sample, an 88.9% of the age group 0-4 was from native origin, a 66.7% presented a visceral type of leishmaniasis, and 85.19% evolved to healing without sequel. Is important to notice that there was no differences between native or foreign-born population in this age group during the three studied periods (Fisher's *p* = 0.142). Moreover, cases in the 0-4 age group during the third studied period (2016–2019), only occurred in Spanish-born population. In the elderly group, there was a rise in the diagnosis of cutaneous leishmaniasis, being 64% of the total cases over 65 years of age. This group only presented a 20% history of immunosuppression and a 24% foreign origin. A 76% of the cases evolved to healing without sequel. The reasons for this increase are unclear and may be related to a change in the diagnosis system and access to dermatological services from primary care, but further investigation is required.

Among population from foreign country of birth, there is a persistent increase of incidence since 2003, consistent with a rise in foreign population migrating to Barcelona (3.8% of the total population in 1996 to 25.7% in 2019) (31), but also to international traveling to endemic areas, due to tourism, visiting relatives and work (22, 36). The difference between autochtonous and imported cases is difficult to establish due to the long incubation period of leishmaniasis (2 weeks to several years), and inconsistencies in the reporting of identified *Leishmania* species. Thus, our data might be overestimating the burden of locally acquired *Leishmania*. The introduction risk supposed by *Leishmania braziliensis* imported cases is thought to be limited due to the reported absence of competent vectors in Europe (7), although a possible competence of *P. perniciosus* to *L. tropica* should be accounted (37–39). Improvements in the registry should allow for the systematic report of the species of *Leishmania* involved in the clinical cases.

In relation to vulnerable populations, there was a decrease in cases related to immunosuppression, due to disease or treatment. This decrease could correspond to the improve of the antiretroviral treatments for HIV population (16), as well as improvements in the care of immunosuppressed patients, like transplanted, who presents a very low incidence of cases. In this study, most of the cases occurred in disadvantaged districts of Barcelona, without changes in time. Homeless cases are at a higher risk to get lost to follow up, facing a challenge to the prevention and surveillance strategy for years to come. Even when we did not have access to information of the individual's socio-economic status, the use of the Available Family Income Index is a good proxy to evaluate inequalities within the city (32).

Leishmaniasis is the most relevant endemic zoonosis in the last years in the Mediterranean basin, due to the coexistence of *Leishmania* protozoa; reservoirs such as hare (11, 40), rabbits (10), rodents and dogs (9, 41), and competent vectors (sandflies from the genus *Phlebotomus)*. As a Mediterranean city, Barcelona has the appropriate climatic characteristics for local transmission of *Leishmania* sp. The presence of the two main vectors, *Phlebotomus perniciosus* and *Phlebotomus ariasi*, are known due to the monitoring of the city of Barcelona by the ASPB, the institution responsible for the vector surveillance and control in the city (42). Furthermore, recent research indicates that Norway rat, a synanthropic murid that shows high abundance and deeply distribution in the sewer system of the city (6), could play a relevant role as host reservoir of Leishmaniasis (43). At this point, it is important to emphasize the sewers have been also recorded as suitable breeding site for sandflies in Barcelona (unpublished data).

Climate change is affecting the geographical distribution and seasonality of many species, including sandflies, and increased connectivity has facilitated the expansion of different insect vectors into new geographical areas (7, 21). A climate change induced expansion of vector competent species will at the same time lead to an expansion of the risk area for leishmaniasis. The expansion of the green areas across the city due to the Climate change action plan (44) should consider the potential risk of increase in competent vector distribution and reservoirs. Overall, there is a need to enforce the surveillance system of leishmaniasis. Therefore, we recommend undertaking surveys in the city to characterize the distribution and abundance of different vectors, as a strategy to establish an effective surveillance and control program to reduce the risk of transmission.

Our results show that that autochthonous transmission is occurring in the city of Barcelona and consequently a more in-depth study of the vectors present in the city should be carried out, in order to know their distribution, abundance, and blood feeding behavior and thus have a diagnosis of the risk of transmission and spillover into humans. Epidemiological, vectorial and health surveillance must have in consideration the high risk groups, like homeless and exposed workers, as those who work in sewer systems, together with vector control programs associated to renovation works affecting the underground structure that may facilitate the movement of *Phlebotomus* into inhabited areas (3).

The main limitations of this study are the loss of follow up of some cases, and the lack of information regarding significant variables. There is a regular loss of cases each year, which may be due to cases that occurred in the homeless, or people that were in transit and the clinical history is incomplete. There is a clear need to review the current epidemiological survey to systematically collect information on the detected leishmania species, the type of treatment used and its efficacy, as well as homelessness status, to better characterize the impact of leishmaniasis on vulnerable populations. Identifying the domestic animals that act as reservoirs and ensuring they receive the necessary treatments to reduce the risk of new transmissions to humans should also be a priority to reduce the risk of local outbreaks (45, 46). It is also important to quantify the role that non-domestic animal species may play as reservoirs associated to human infection cases in the city (5, 47). Collaboration at an epidemiological and entomological level is one of the pillars to reduce the risks of transmission, thus trying to work on a One Health concept that integrates the different fields involved in the transmission processes.

Even though leishmaniasis is an endemic disease in Spain, the city of Barcelona will continue to be a highly transited city, with a higher migration rate and tourism, which may favor the occurrence of imported cases. This constitutes an entomological, clinical, and epidemiological challenge, due to the variety of clinical presentations and adaptation to vectors or local reservoirs.

## Data Availability Statement

The raw data supporting the conclusions of this article will be made available by the authors, without undue reservation.

## Ethics Statement

Ethical review and approval was not required for the study on human participants in accordance with the local legislation and institutional requirements. Written informed consent from the participants' legal guardian/next of kin was not required to participate in this study in accordance with the national legislation and the institutional requirements.

## Author Contributions

TM, J-PM, and CR worked in the conception and first design of the work. LM, PS, and EM were in charge of the acquisition of the data, and organization of the database. DP performed the statistical analysis and wrote the first draft of the manuscript. DP, LM, and JF worked in the interpretation of data for the work. DP and LM were in charge of drafting the work. RB-M, JF, PS, EM, CR, and TM critically revised the manuscript. All authors provide their final approval of the version to be published and agreed to be accountable for all aspects of the work in ensuring that questions related to the accuracy or integrity of any part of the work are appropriately investigated and resolved.

## Conflict of Interest

The authors declare that the research was conducted in the absence of any commercial or financial relationships that could be construed as a potential conflict of interest.
