# Supplementary material for: Trends in the Epidemiology of Leishmaniasis in the City of Barcelona (1996–2019)
Source: Front Vet Sci. 2021 Apr 26;8:653999. doi: 10.3389/fvets.2021.653999 (PMC8107217; doi:10.3389/fvets.2021.653999)
Supplement: Supplementary file 1 [file Table_1.DOC]

**Annex 1**

Novembre 2015


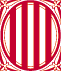
Departament de Sanitat Codi reservat a la regió sanitària

i Seguretat Social

**Direcció General**

Generalitat de Catalunya Malatia Any Regió Num. fitxa

Fitxa epidemiològica. **Cas de Leishmaniosi**

**Dades del pacient**

Nom Cognoms

Data de naixement Sexe: Home Dona

Adreça Tel:

Municipi Província DM

País d'origen

(Si resideix a l'estranger, especifiqueu-ne el país)

Data d'inici dels símptomes

**Dades del metge declarant**

Nom, cognoms Núm. coleg

Centre sanitari Codi Tel

Municipi Província Codi

Data declaració Setmana declaració

**Dades clíniques i diagnòstiques**

Data del diagnòstic

Hospitalització: Sí No Data hospitalització Centre

Tipus leishmaniosi: Visceral Cutània cutània mucosa

**Dades de laboratori**

1 Biòpsia  1 Positiu  2 Negatiu

2 Cultiu  1 Positiu  2 Negatiu

3 Serologia  1 Positiu  2 Negatiu Especifiqueu-ne la tècnica

**Dades epidemiològiques** (acoteu les preguntes a un període màxim de dos anys)

1. Heu tingut contacte amb un gos malalt o amb un altre animal?  1. Sí  2. No

(rosegadors, marsupials)

2. Heu viatjat a una àrea endèmica?  1. Sí  2. No

En cas afirmatiu, especifiqueu-ne el país

(Índia, Paquistan, Orient M, Sud Rússia, litoral mediterrani,

Àfrica, Texas, Mèxic, Sud Amèrica)

3. Altres factors de risc  1. Sí  2. No

(interrogueu sobre contactes amb insectes vectors, flebotomus)

En cas afirmatiu, especifiqueu-los

4. Teniu antecedents de malaltia immunosupressora?  1. Sí  2. No

En cas afirmatiu especifiqueu-la

5. Han fet tractament immunodepressor?  1. Sí  2. No

6. Sou addicte a drogues per via parenteral?  1. Sí  2. No

7. Heu rebut alguna transfusió?. En cas afirmatiu:  1. Sí  2. No

Lloc Data

8. Teniu antecedent de transplantament?  1. Sí  2. No

9. I d'alcoholísme?  1. Sí  2. No

10. Heu tingut contacte sexual amb alguna persona infectada?  1. Sí  2. No

**Activitats de control**

S'han pres mesures de control?  1. Sí  2. No

En cas afirmatiu, especifiqueu-les

**Dades evolutives**

 1. Curació sense seqüeles

 2. Curació amb seqüeles

 3. Defunció

 4. No cas

 5. Perdut

Estat cas: Cas sospitós  Cas confirmat  No cas 

**Observacions**

Enquestador/a Tel Data tancament

**Annex 2:**

Generalitat de Catalunya. Departament de Salut

**Definition of cases of notifiable diseases – Leishmaniasis[[1]](#footnote-2)**

**Clinical Description**

- **Cutaneous and mucocutaneous leishmaniasis**

Cutaneous leishmaniasis or oriental ulcer is a disease caused by leishmania (*Leishmania infantum and Leishmania tropica*) and transmitted by the sting of a dipteran of the genus *Phlebotomus* that is characterized by long-lasting, single or more rarely multiple painless ulcerative lesions.

Mucocutaneous or spongy leishmaniasis, caused by several species of American *Leishmania* and transmitted by the sting of a dipteran of the genus *Lutzomyia*, is manifested by the same skin lesions and, in addition, mutilating ulcerative lesions in the pharynx and nose.

- **Visceral leishmaniasis or kala-azar**

Disease caused by leishmania (*Leishmania infantum* and *Leishmania donovani*, and *Leishmania chagasi* in America) and transmitted by the sting of a dipteran of the genus *Phlebotomus* in the old world and *Lutzomyia* in America which is characterized by fever with alternating periods of apyrexia, hepatosplenomegaly which can become very important, pancytopenia, hypergammaglobulinemia and emaciation accompanied by progressive weakness

**Laboratory criteria for diagnosis**

- **Cutaneous and mucocutaneous leishmaniasis.** One of the two:

❐ Isolation of the causative agent.

❐ Identification of leishmania amastigotes by microscopic examination of stained material obtained by aspiration or scraping, from the edges of the lesions

- **Visceral leishmaniasis or kala-azar:** One of the two:

❐ Isolation of the causative agent.

❐ Observation of amastigotes in smears stained with material obtained from bone marrow, spleen, lymph nodes and blood.

❐ Positive serology by ELISA or indirect immunofluorescence.

**Case confirmed:** Clinically compatible disease, laboratory confirmed.

**Suspicious case:** Clinically compatible disease.

1. https://canalsalut.gencat.cat/web/.content/_Professionals/Vigilancia_epidemiologica/documents/arxius/spdefimdo.pdf [↑](#footnote-ref-2)
